# Efficient suppression of back electron/hole recombination in cobalt phosphate surface-modified undoped bismuth vanadate photoanodes†
†Electronic supplementary information (ESI) available: XRD characterization and SEM images of CoPi-modified and unmodified BiVO_4_, continuous illumination *J*–*V* curves, transient absorption fit results, and transient absorption decays measured as a function of excitation intensities. See DOI: 10.1039/c5ta05826k



**DOI:** 10.1039/c5ta05826k

**Published:** 2015-09-21

**Authors:** Yimeng Ma, Florian Le Formal, Andreas Kafizas, Stephanie R. Pendlebury, James R. Durrant

**Affiliations:** a Department of Chemistry , Imperial College London , South Kensington Campus , London , SW7 2AZ , UK . Email: j.durrant@imperial.ac.uk; b Laboratory for Molecular Engineering of Optoelectronic Nanomaterials , Institute of Chemical Sciences and Engineering , École Polytechnique Fédérale de Lausanne (EPFL) , Station 6 , CH H4 565 , Lausanne 1015 , Switzerland

## Abstract

In this paper, we compared for the first time the dynamics of photogenerated holes in BiVO_4_ photoanodes with and without CoPi surface modification, employing transient absorption and photocurrent measurements on microsecond to second timescales. CoPi surface modification is known to cathodically shift the water oxidation onset potential; however, the reason for this improvement has not until now been fully understood. The transient absorption and photocurrent data were analyzed using a simple kinetic model, which allows quantification of the competition between electron/hole recombination and water oxidation. The results of this model are shown to be in excellent agreement with the measured photocurrent data. We demonstrate that the origin of the improvement of photocurrent onset resulting from CoPi treatment is primarily due to retardation of back electron/hole recombination across the space charge layer; no evidence of catalytic water oxidation *via* CoPi was observed.

## Abstract 2

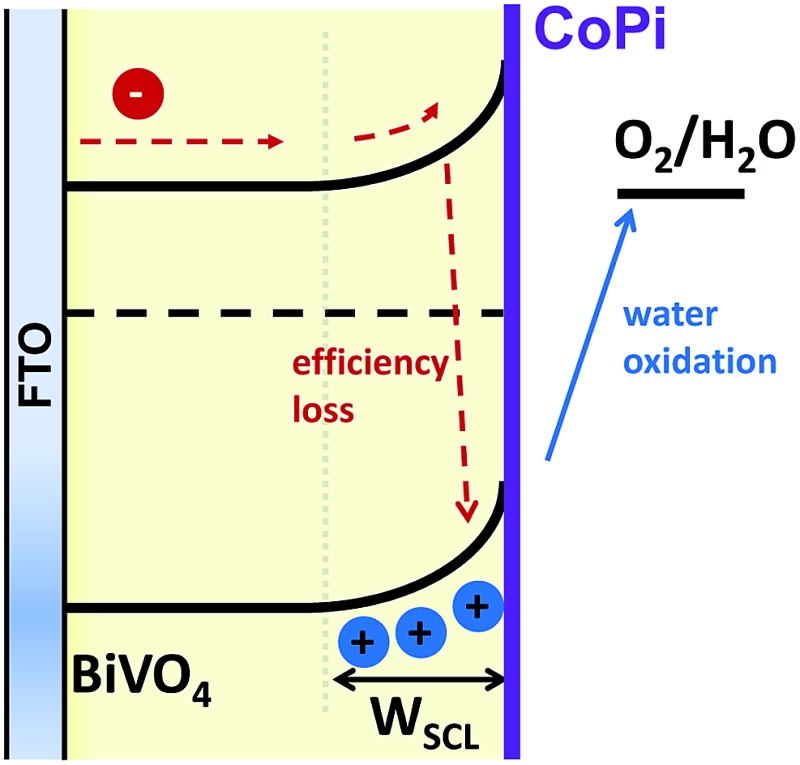
BiVO_4_/CoPi photoanodes are studied using transient absorption spectroscopy and transient photocurrent methods. The cathodic shift of photocurrent onset potential is due to efficient suppression of back electron/hole recombination on the timescale of seconds.

## 1. Introduction

Surface modification of n-type semiconductor photoanodes is an effective method to promote photo-assisted water oxidation.^1^ Such surface modifications include the deposition of both catalytic (*e.g.* cobalt oxides) and non-catalytic layers (*e.g.* Al_2_O_3_) on photoanodes such as hematite (α-Fe_2_O_3_)^2–7^ and bismuth vanadate (BiVO_4_).^8–12^ However, the mechanism by which such surface modifications enhance photoanode function remains unclear.^13^ In particular it is unclear whether the electrocatalytic properties of the surface layer with respect to water oxidation are functionally important. Whilst several studies have suggested that enhancements in photoanode performance are associated with the catalytic properties of the surface modification layer,^8,10,14^ we,^4,5^ and others,^15^ have reported evidence that, at least for cobalt phosphate (CoPi) modified hematite, the enhancement in photoanode performance results primarily from a retardation of recombination kinetics. These studies indicated that the kinetics of water oxidation on hematite are unchanged by CoPi modification.

Bismuth vanadate (BiVO_4_) is a promising photoanode material for photoelectrochemical (PEC) water splitting,^1,16,17^ where surface modifications can be particularly effective in shifting the photocurrent onset close to the flat-band potential. Surface modification with CoPi electrocatalysts has resulted in a shift of the onset potential for photocurrent generation from ∼0.8 *V*_RHE_ to 0.4 *V*_RHE_.^10^ Additionally, a NiOOH/FeOOH dual layer surface modified BiVO_4_ has been reported with the onset potential (0.2 *V*_RHE_) close to the flat-band potential (0.1 *V*_RHE_).^18^ These reports clearly demonstrate the importance of surface modification using electrocatalysts if high energy conversion efficiencies are to be achieved at low applied potentials.^8,18,19^ In the study reported herein, we employ transient absorption and transient photocurrent measurements to investigate the origin of such cathodic shifts of photocurrent onset potential, employing undoped BiVO_4_ photonodes with and without CoPi surface modification.

Recently, we investigated the charge carrier dynamics of photogenerated holes in flat, dense, undoped BiVO_4_ photoanodes using transient absorption spectroscopy and photoelectrochemical methods.^20^ We observed that the yield of long-lived photogenerated holes increased with increasing space charge layer depth. However, the efficiency of water oxidation was found to be severely limited by recombination between these long-lived holes accumulated at the BiVO_4_ surface and electrons in the BiVO_4_ bulk. Due to the rather slow rate constant for water oxidation (∼1 s^–1^), there is significant kinetic competition between this back electron/hole recombination and water oxidation at moderate applied bias potential. Consequently, approximately 600 mV additional anodic potential is required for photocurrent generation, due to this slow surface recombination. It is therefore of particular interest to consider whether surface treatments impact upon this kinetic competition by retarding back electron/hole recombination and/or by accelerating water oxidation.

CoPi was first reported as an efficient water oxidation electrocatalyst by Kanan and Nocera, significantly reducing the overpotential for water oxidation.^21^ X-ray absorption studies indicate that the structure of CoPi composite is amorphous, forming a cubane-like CoO_*x*_ reaction center with phosphate ligands.^22–24^ CoPi is also widely reported to reduce the photocurrent onset potential and increase the photocurrent of photo-assisted water oxidation on BiVO_4_ (ref. 8 and 11) and α-Fe_2_O_3_.^4,7^ It was initially suggested that photo-driven water oxidation in these systems occurred *via* oxidation of the CoPi catalyst by photogenerated holes from the semiconductor. This CoPi oxidation process also implies a charge separation at the semiconductor surface, which could mitigate electron/hole recombination thought to occur *via* an intra-band gap state at the surface.^8,14,25^ This conclusion of enhanced charge separation due to hole transfer has been supported by studies comparing water oxidation photocurrents for photoanodes with and without CoPi modification in a hole scavenger solution.^8,10^ However, our previous transient optical studies of CoPi-modified α-Fe_2_O_3_ indicated that the most significant effect of CoPi is to reduce the anodic potential required to retard recombination losses within the α-Fe_2_O_3_. This reduces the bias potential required to generate the long-lived holes required for water oxidation.^4,5^ Whilst steady-state CoPi oxidation was observed, it was suggested that this oxidation was not the primary reason for the cathodic shift of the photocurrent onset potential.^4^ A similar conclusion was also obtained from frequency domain photoelectrochemical analyses.^15^ It is not clear, however, whether this observation is specific to the CoPi surface modified α-Fe_2_O_3_ system, or a general effect for n-type semiconductors surface-modified by CoPi. In addition, our previous studies of CoPi-modified α-Fe_2_O_3_ did not distinguish between the impact of CoPi upon bulk recombination losses (which limit hole transfer to the surface) or upon back electron/hole recombination. This latter (slower) recombination pathway is particularly important in limiting the onset potential for undoped BiVO_4_ photoanodes.

In the study reported herein, we employ transient absorption spectroscopy (TAS) and transient photocurrent (TPC) measurements to investigate the time-resolved behavior of photogenerated holes on CoPi-modified BiVO_4_ photoanodes. Transient absorption spectroscopy allows us to directly monitor photogenerated holes. The recombination and water oxidation kinetics occurring on CoPi-modified BiVO_4_ were studied as a function of applied potential. The origin of the improvement of photocurrent onset resulting from CoPi treatment is shown to be primarily due to a retardation of back electron/hole recombination across the space charge layer; no evidence of catalytic water oxidation *via* CoPi was observed.

## 2. Experimental section

### 2.1 Materials

All chemicals used in this paper were purchased from Sigma-Aldrich in the highest purity available, unless otherwise stated. The electrolyte used was prepared with de-ionized Milli-Q water (Millipore Corp., 18.2 M Ω cm at 298 K).

### 2.2 Fabrication of undoped BiVO_4_ photoanodes

Undoped BiVO_4_ photoanodes were fabricated using a modified metal-organic deposition method, as previously described.^20,26^ Briefly, the precursor was prepared by mixing 0.2 M bismuth nitrate pentahydrate dissolved in acetic acid (99%, BDH) and 0.2 M vanadyl acetylacetonate in acetylacetone (Merck) for 1 hour at room temperature. The BiVO_4_ photoanodes were deposited using the precursor on FTO substrates (TEC 15, Hartford Glass Co.) by the spin-coating method (1000 rpm, 20 s). After spin-coating each layer, the substrates were calcined at 450 °C for 15 min. The total number of layers was 15 for each BiVO_4_ photoanode and the films were calcined at 450 °C for 5 hours.

### 2.3 Photoelectrodeposition of CoPi on undoped BiVO_4_ photoanodes

Photoelectrodeposition of CoPi on undoped BiVO_4_ photoanodes were carried out following the method developed by Nocera^21^ and modified by Gamelin.^7,8^ A three-electrode configuration was used, including the undoped BiVO_4_ photoanode as the working electrode, a Ag/AgCl/sat'd KCl as the reference electrode and a platinum mesh counter electrode. The deposition solution was prepared with 0.1 mM cobalt nitrate hexahydrate in 0.1 M KPi buffer (50 mL, pH 6.7). The deposition was carried out at 1.2 *V*_RHE_ applied potential by an Autolab potentiostat (PGSTAT101, Metrohm) and under illumination from a Xe lamp coupled with a KG3 filter for 10 minutes; intensity calibrated with a Si photodiode corresponding to 100% of the AM 1.5 solar spectrum in terms of photo flux below 650 nm (with a short pass filter, 650 nm, Edmund Optics).

### 2.4 Characterization

X-ray diffraction (XRD) was conducted with a modified Bruker-Axs D8 diffractometer with parallel beam optics equipped with a PSD Linx-Eye silicon strip detector. A Cu source generated X-rays; with Cu Kα1 and Cu Kα2 radiation of *λ* = 1.54056 and 1.54439 Å, respectively, emitted with an intensity ratio of 2 : 1 at an applied potential of 40 kV and 30 mA of current. The incident beam was kept at 1° and the angular range of the patterns collected was 10° < 2*θ* < 66° with a step size of 0.025°.

Scanning electron microscopy (SEM) measurements were carried out using a ZEISS FEG-SEM microscope (LEO 1525 equipped with GEMINI field emission column) with acceleration voltage of 5 kV. Samples prepared for SEM measurements were coated with 10 nm chromium particles to enhance conductivity of BiVO_4_ films.

### 2.5 Photoelectrochemical characterization

All original applied potentials are recorded *versus* Ag/AgCl/sat'd KCl reference electrode (0.197 *V*_NHE_ at 298 K; Metrohm). These potentials are then converted to be *versus* reversible hydrogen electrode using Nernst equation:

1 *V*_RHE_ (V) = *V*_Ag/AgCl_ (V) + 0.0591 × pH + *V*0Ag/AgCl

where *V*_RHE_ is the applied potential *versus* RHE; *V*_Ag/AgCl_ (V) is the applied potential *versus* Ag/AgCl/sat'd KCl reference electrode during measurements; *V*0Ag/AgCl is the standard potential of the Ag/AgCl reference electrode.

Photoelectrochemical measurements were carried out in a home-made PTFE cell with quartz windows in the front and back of the cell. The same three-electrode setup was used as described in the CoPi deposition section. All PEC experiments were carried out through back-side illumination (FTO-BiVO_4_) in order to avoid the parasitic absorption by CoPi. An Autolab potentiostat (PGSTAT 12) was used to control the applied potential. Potassium phosphate buffer solution (0.1 M K_2_HPO_4_ and 0.1 M KH_2_PO_4_) was prepared as the electrolyte for PEC and transient absorption measurements.

Transient photocurrent measurements were carried out with the same photoelectrochemical cell as for PEC measurements. A constant anodic potential was applied to the BiVO_4_ working electrode and the current response was measured under chopped light illumination from a 365 nm LED (LZ1-10U600, LedEngin Inc.). The LED intensity was adjusted to be 20% of AM 1.5 intensity below 500 nm (*i.e.* BiVO_4_ absorption edge). In order to achieve small perturbation excitation, a continuous bias light was employed using an array of 12 white LEDs (Philips Lumileds, Model white star LXHL-NWE8) with ∼100% of AM 1.5 intensity below 650 nm, calibrated with the Si photodiode. The chopped light was controlled by a chopper with time interval of 5 s.

### 2.6 Transient absorption spectroscopy

The setup of transient absorption spectrometer (μs–s) has been described previously.^20^ Briefly, a Nd:YAG laser (Big Sky Laser Technologies, Ultra CFR Nd:YAG laser system) was used as the excitation source (355 nm, 3^rd^ harmonic, pulsed laser, 6 ns band width). The laser flash rate was 0.33 Hz; the laser excitation intensity was set at 100 μJ cm^–2^, unless otherwise stated; the probe light source was a 100 W Bentham IL1 tungsten lamp equipped with a monochromator (OBB-2001, Photon Technology International); the transmitted light was filtered using long pass filters and a band pass filter (Comar Optics) to block the scattered laser light into the detector (Si photodiode, Hamamatsu S3071). The signal collected by the detector was sent to an amplifier (Costronics) and recorded by an oscilloscope (Tektronics TDS 2012c) on μs–ms timescales and a DAQ card (National Instruments, NI USB-6211) on ms–s timescales. Each decay was averaged by 300–500 times. All data were acquired by home-programmed software based on the LabVIEW software.

## 3. Results

Flat, 450 nm thick undoped BiVO_4_ films were fabricated by spin-coating using the metal-organic deposition method previously reported.^20,26^ We have reported material characterizations of these films previously (XRD, SEM, UV-vis).^20^ CoPi was photo-electrodeposited on these BiVO_4_ photoanodes according to methods developed previously,^7,8,21^ with the thickness of the CoPi overlayer ∼100 nm being selected to give optimum performance enhancement (ESI Fig. S1†). The flat BiVO_4_ photoanodes requires thicker CoPi overlayers than reported 30 nm for porous BiVO_4_ photoanodes.^11^ This result is similar to a previous report of CoPi-modified α-Fe_2_O_3_ nanostructured/flat photoanodes.^6^ X-ray diffraction characterization in Fig. S1† indicates that the CoPi deposited on BiVO_4_ is amorphous, consistent with previous reports of CoPi overlayers prepared *via* electrochemical methods.^21–23^

**Fig. 1:**
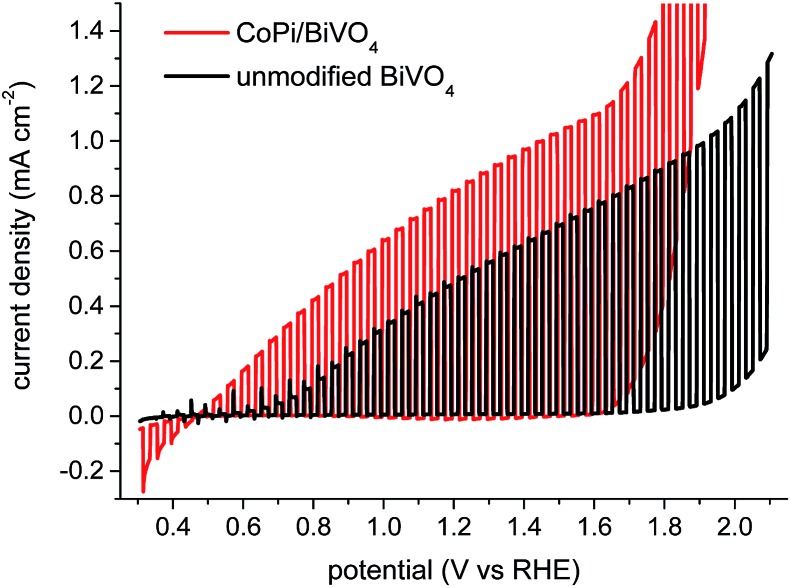
Chopped light *J*–*V* curve for unmodified (black) and CoPi-modified (red) BiVO_4_ photoanodes in 0.1 M KPi buffer (pH 6.7), under back-side illumination (FTO-BiVO_4_); the *J*–*V* curve under continuous illumination is shown in ESI Fig. S2.† The scan rate is 10 mV s^–1^ and the chopping frequency is 0.2 Hz.

Fig. 1 compares the chopped light current–voltage of BiVO_4_ photoanodes before and after CoPi modification, measured in potassium phosphate buffer (KPi, pH 6.7). For the unmodified BiVO_4_ photoanodes, the onset potentials (*versus* reversible hydrogen electrode, RHE) for water oxidation in light and dark are 0.7 *V*_RHE_ and 1.9 *V*_RHE_, respectively, consistent with our previous studies^20^ and literature data.^1,10,18,26^ A cathodic shift of 200–300 mV for the onset potentials of both dark and light current generation is observed for the CoPi-modified BiVO_4_ photoanode, similar to previous reports.^8,11,27^ The more cathodic dark onset obtained with the CoPi overlayer is consistent with its electrocatalytic properties.^21,23,28^

**Fig. 2:**
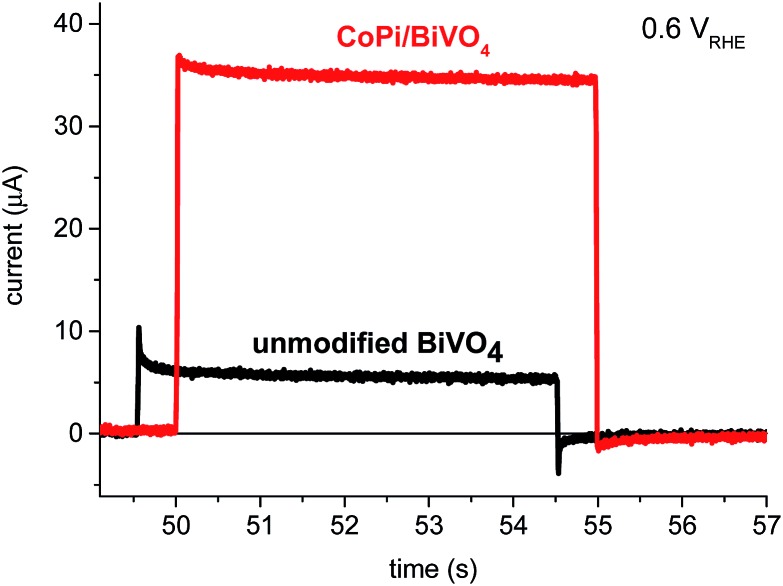
Transient photocurrent measured at 0.6 *V*_RHE_ for unmodified (black) and CoPi-modified (red) BiVO_4_ photoanodes under white light bias illumination with a 5 s small-perturbation 365 nm pulse. The background currents due to bias light have been corrected to zero.

Transient photocurrent (TPC) measurements (using white light bias and a 5 s small-perturbation 365 nm LED pulse) were conducted as an initial study of charge carrier dynamics. Fig. 2 presents typical TPC results for unmodified (black) and CoPi-modified (red) BiVO_4_ photoanodes under modest applied anodic potential (0.6 *V*_RHE_), just anodic of the photocurrent onset. We are particularly interested in the negative transient current peaks observed after light off, assigned to back electron/hole recombination.^20,29,30^ This negative transient is reduced in amplitude and retarded in recovery time in the presence of the CoPi overlayer, indicative of suppression of back electron/hole recombination,^30^ discussed further below.

Transient absorption spectroscopy was employed to investigate the dynamics of photogenerated holes in CoPi-modified BiVO_4_ water oxidation photoanodes. Previously we have shown that the transient absorption spectrum of photogenerated holes in unmodified BiVO_4_ photoanodes comprises a broad absorption across the visible and near-IR, with a maximum at 550 nm, which was also reported by other groups.^31,32^ Under anodic bias, the decay dynamics of this photoinduced absorption for an unmodified undoped BiVO_4_ photoanode were observed to be biphasic. A “fast” power law phase, assigned to electron/hole recombination competing with hole accumulation on the BiVO_4_ surface, is observed on μs–ms timescales. This is followed by a “slow” exponential decay phase (ms–s) assigned to water oxidation in kinetic competition with back electron/hole recombination.^20^

**Fig. 3:**
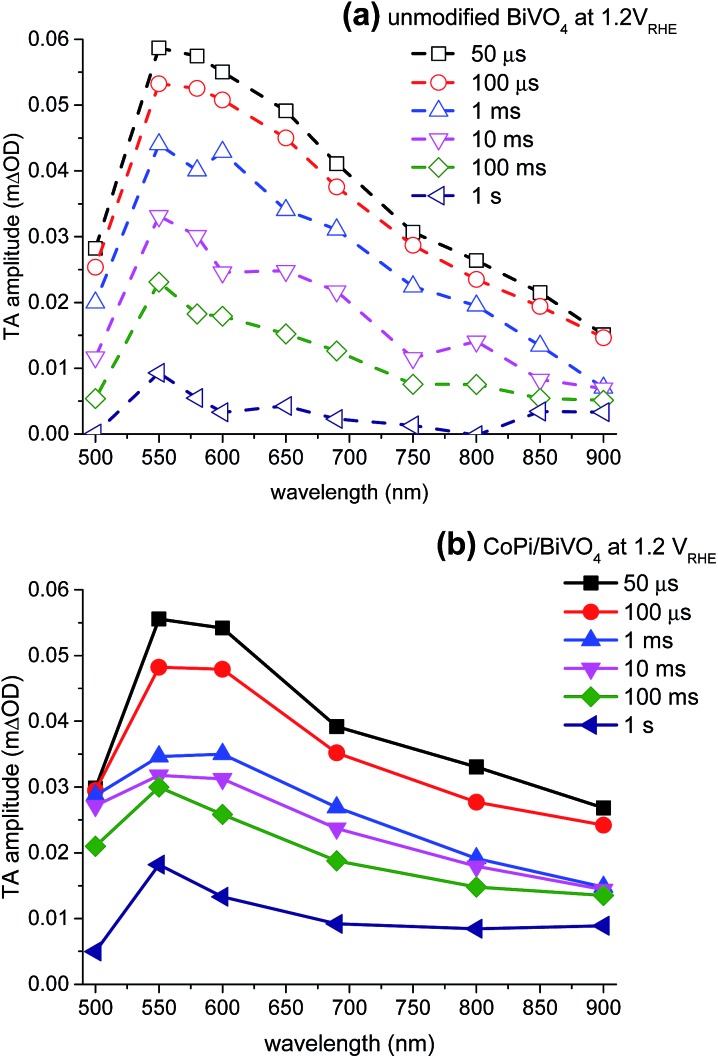
Transient absorption spectra of (a) CoPi-modified BiVO_4_ (solid lines and filled symbols) and (b) unmodified BiVO_4_ (dashed lines and empty symbols) at 1.2 *V*_RHE_, recorded from 50 μs to 1 s after laser excitation (355 nm, ∼100 μJ cm^–2^, 0.33 Hz).

We first compare the transient absorption spectra of unmodified BiVO_4_ and CoPi-modified BiVO_4_, from microsecond to second timescales, at 1.2 *V*_RHE_. Fig. 3a shows the transient absorption spectra of unmodified BiVO_4_ at 1.2 *V*_RHE_ applied potential. A broad transient absorption feature was observed between the timescale of microseconds and seconds, from 500 nm to 900 nm, peaking at 550 nm, consistent with our previous transient absorption results of unmodified BiVO_4_ with applied potential. These transient absorption spectra have been assigned to photogenerated holes in unmodified BiVO_4_, as determined using electron/hole scavengers and applied potentials.^20^ We have previously shown that the shape of the transient absorption spectrum of photogenerated holes in BiVO_4_ is independent of applied potential.^20^ Fig. 3b shows that CoPi surface modification does not significantly change the shape or the amplitude of the transient absorption spectra; both photoanodes exhibit broad spectra peaking at 550 nm with initial amplitudes of ΔOD ∼5.5–6 × 10^–5^. We note that CoPi oxidation results in a broad increase of the steady-state absorption in the visible region, particularly at wavelengths <550 nm.^4^ This is clearly different from the transient absorption spectra (Fig. 3). Therefore we assign the transient absorption signals for both the CoPi-modified and unmodified BiVO_4_ primarily to photogenerated BiVO_4_ holes.

**Fig. 4:**
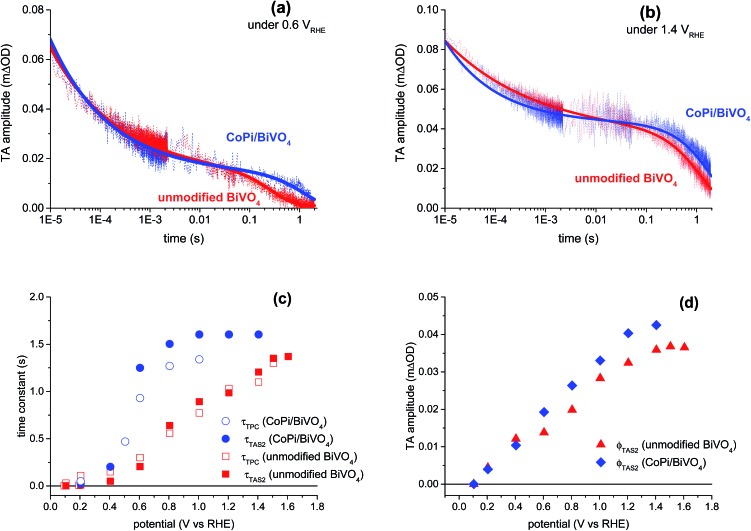
(a) and (b) Comparison of transient absorption decays of unmodified BiVO_4_ (red) and CoPi-modified BiVO_4_ (blue) photoanodes measured in 0.1 M KPi buffer under 0.6 *V*_RHE_ (a) and 1.4 *V*_RHE_ (b). Fits to the data using eqn (2) (blue lines: CoPi-modified BiVO_4_; red lines: unmodified BiVO_4_) are overlaid with the measured transient absorption data. (c) Time constants from transient absorption and transient photocurrent decays *versus* applied potential. Filled circles and squares: *τ*_TAS2_, time constants from the exponential decay phase (eqn (2)) for unmodified (red) and CoPi-modified (blue) BiVO_4_ photoanodes. Empty circles and squares: *τ*_TPC_, the time constants of negative transient current, for unmodified (red) and CoPi-modified (blue) BiVO_4_ photoanodes, in the TPC measurements, obtained from fits using eqn (3). (d) Amplitude of the slow transient absorption decay phase *φ*_TAS2_ *versus* applied potential; red triangles: unmodified BiVO_4_; blue diamonds: CoPi-modified BiVO_4_.

Fig. 4 shows typical transient absorption decay kinetics of photogenerated holes measured at 550 nm for both unmodified and CoPi-modified BiVO_4_ photoanodes under modest (0.6 *V*_RHE_) and strong (1.4 *V*_RHE_) anodic potentials. As previously,^20,33,34^ these decays were fitted by a combination of power law (for the “fast” decay phase) and single-exponential (for the “slow” decay phase) functions,

2 ΔOD = *at*^*b*^ + *φ*_TAS2_e^–*t*/*τ*_TAS2_^

where *a* and *b* define the power law function, *φ*_TAS2_ is the amplitude of the exponential decay phase and *τ*_TAS2_ is its decay time constant. The power law represents a bimolecular recombination process on sub-milliseconds timescales, as determined by transient absorption studies of unmodified BiVO_4_ as a function of laser excitation intensity.^20^ As previously demonstrated for unmodified BiVO_4_, both water oxidation and back electron/hole recombination occur on timescales of milliseconds to seconds,^20^ fitted by the single exponential function in eqn (2).

It is apparent from Fig. 4a and b that, under the low applied potential, the lifetime of the exponential decay is increased by up to one order of magnitude after CoPi-modification. Similar results were obtained from our transient absorption studies of CoPi surface-modified α-Fe_2_O_3_ photoanodes.^4,5^ Considering both the transient absorption spectra (Fig. 3) and the kinetics (Fig. 4), there is no evidence for hole transfer from BiVO_4_ to CoPi under these pulsed laser measurements. If hole transfer was occurring from BiVO_4_ to CoPi (as would be expected if water oxidation occurs *via* CoPi), this would result in changes to the spectrum and faster decay kinetics. Neither of these phenomena are observed. These results suggest that, for the films studied herein, water oxidation does not primarily occur *via* hole transfer from BiVO_4_ to CoPi, in contrast to the conclusion of previous photoelectrochemical studies.^8,10^ Instead, the primary effect of CoPi overlayers is to slow down electron/hole recombination within the BiVO_4_, as demonstrated below.

Fig. 4c and d show the lifetimes and amplitudes of the slow exponential decay phase in unmodified and CoPi-modified BiVO_4_ as a function of applied anodic potential, determined from fits of the data to eqn (2). The decay time constants and amplitudes increase with increasing anodic potential in both photoanodes. It is apparent that CoPi deposition does not significantly change the amplitude of the exponential decay phase, *φ*_TAS2_, nor its dependence upon applied potential (Fig. 4d). In contrast, the potential dependence of the lifetime of this decay phase (*τ*_TAS2_) is substantially different after CoPi deposition (Fig. 4c). For unmodified BiVO_4_, *τ*_TAS2_ increases gradually across the potential range studied, from ∼10 ms at 0.2 *V*_RHE_ to ∼1.4 s at 1.6 *V*_RHE_. In contrast, for the CoPi-modified photoanode, *τ*_TAS2_ increases in lifetime much more sharply with applied potential, saturating at 1.6 s for potentials ≥1.0 *V*_RHE_. We have previously assigned the potential dependence of *τ*_TAS2_ for unmodified BiVO_4_ to retardation of back electron/hole recombination.^20^ The significant increase in lifetime of the ms–s exponential phase under modest applied potentials following CoPi deposition suggests that this back electron/hole recombination is retarded by CoPi deposition, as we discuss in more detail below.

To provide further evidence of retardation of back electron/hole recombination on BiVO_4_ by CoPi deposition, transient photocurrent data such as that shown in Fig. 2 were collected as a function of applied potential from 0.1 to 1.6 *V*_RHE_. The positive and negative current transients immediately after light on/off are associated with recombination of surface accumulated holes and bulk electrons. In addition, there is a contribution from water oxidation to the negative transient after light off. These transients are well fitted by a single exponential function.^2,20,29,30^ In this paper, we are particularly interested in the negative current transients after light off. The background current due to the bias light was subtracted, and the negative transient peaks were fitted by a single exponential function:

3 *I*(*t*) = *I*_0_e^–(*t*/*τ*_TPC_)^

where *I*(*t*) is the transient current measured under applied potential; *I*_0_ is the amplitude of negative transient peak, and *τ*_TPC_ is the time constant of the negative transient recovery. We and others have previously assigned this negative current transient to back electron/hole recombination, in kinetic competition with water oxidation.^20,30^ The TPC time constants *τ*_TPC_ are shown in Fig. 4c as a function of applied potential, overlaid with the transient absorption time constants *τ*_TAS2_. There is a good agreement between *τ*_TPC_ and *τ*_TAS2_ for both the unmodified and CoPi-modified BiVO_4_ photoanodes, suggesting that both *τ*_TPC_ and *τ*_TAS2_ are monitoring the same process, consistent with our previous results for unmodified BiVO_4_ photoanodes.^20^

**Fig. 5:**
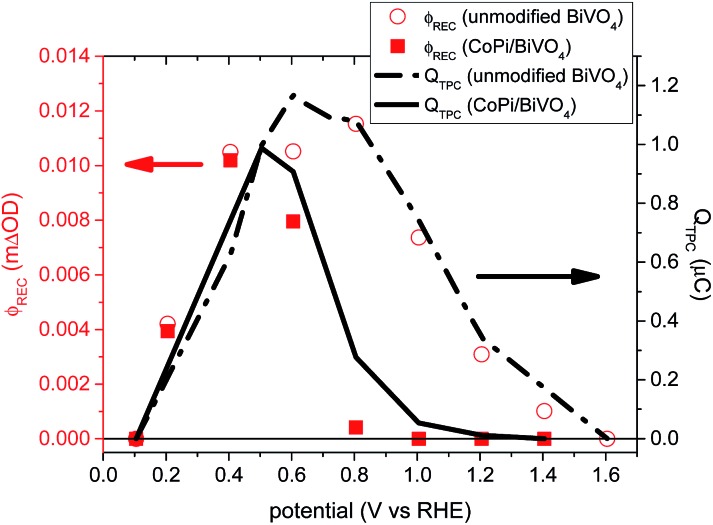
Comparison of back electron/hole recombination loss measured by transient absorption spectroscopy (*φ*_rec_), in unmodified (red empty circles) and CoPi-modified (red filled squares) BiVO_4_ photoanodes, and by TPC measurements (*Q*_TPC_), in unmodified (black dashed line) and CoPi-modified (black solid line) BiVO_4_ photoanodes.

Quantification of the total charge lost to back electron/hole recombination following light-off in our TPC measurement can be obtained by integrating the negative TPC transient. Fig. 5 shows this integrated charge (broken and solid lines) as a function of applied potential for unmodified and CoPi-modified BiVO_4_ photoanodes. The amount of charge (*i.e.* surface accumulated holes) lost due to this back electron/hole recombination is strongly potential-dependent, being maximal at 0.5–0.6 *V*_RHE_. It is also apparent that deposition of CoPi results in a substantial cathodic shift in the potential required to turn off these back electron/hole recombination losses. The disappearance of negative current transients in CoPi-modified BiVO_4_ has also been reported previously.^11^ This cathodic shift of the potential required to turn off back electron/hole recombination is also consistent with our transient absorption data, and likely to be a key reason for the cathodic shift of photocurrent onset potential following CoPi deposition, discussed in detail below.

## 4. Discussion

### 4.1 Correlating long-lived holes with photocurrent density

Water oxidation on metal oxide photoanodes appears to be kinetically rather a slow process.^35^ We have previously reported rate constants for water oxidation under pulsed laser excitation of 0.7 s^–1^ and 5 s^–1^ for hematite^29^ and titania^33^ photoanodes respectively. Similarly slow rate constants have also been reported from electrochemical analyses of hematite photoelectrodes.^15^ For unmodified BiVO_4_, we have reported a rate constant for water oxidation of *circa* 1 s^–1^.^20^ This rate constant was determined from the time constant of the slow decay phase observed by transient absorption under strong anodic bias, where back electron/hole recombination is turned off. This rate constant is consistent with the data reported herein (∼0.7 s^–1^) for our unmodified films at >1.6 *V*_RHE_. Due to these slow rate constants, long-lived holes accumulate at the photoanode surface. Determining the yields and lifetimes of these surface holes is therefore central to understanding the efficiency of water oxidation by such photoanodes.

**Fig. 6:**
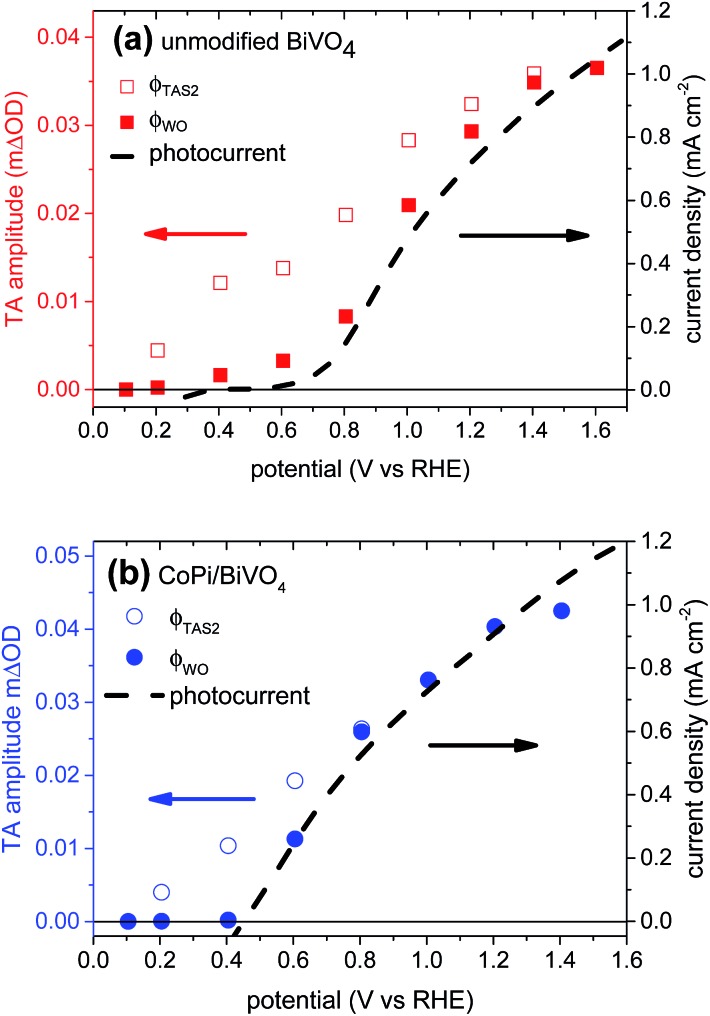
Empty red squares and empty blue circles: transient absorption amplitude of long-lived holes (*φ*_TAS2_) in unmodified (a) and CoPi-modified (b) BiVO_4_ photoanodes, obtained from eqn (2), as a function of applied potential. Filled red squares and filled blue circles: amplitude of holes that contribute to water oxidation (*φ*_WO_) in unmodified (a) and CoPi-modified (b) BiVO_4_ photoanodes, calculated from eqn (4). The photocurrent data (dashed black lines) shown in these figures are directly compared in Fig. S2 in the ESI.†

Our studies herein are focused upon the effect of a photo-electrodeposited surface layer of CoPi electrocatalyst on undoped BiVO_4_ photoanodes. We first consider the impact of CoPi surface modification on the yield of long-lived holes, as assayed by the amplitude *φ*_TAS2_ of the exponential slow phase observed in our transient absorption studies. This yield is a measure of the efficiency of hole transport to the photoanode surface, and is therefore a measure of the efficiency of charge separation, driven primarily by the space charge layer. Fig. 6 (open symbols) shows *φ*_TAS2_ for unmodified and CoPi-modified BiVO_4_ photoanodes as a function of applied potential, overlaid with the photocurrent density. It is apparent that for both photoanodes, there is an offset between the appearance of long-lived photogenerated holes and the photocurrent onset. This difference in the potential dependence of *φ*_TAS2_ and photocurrent is much smaller following CoPi deposition. For unmodified BiVO_4_, we have previously assigned this difference in the potential dependence of *φ*_TAS2_ and photocurrent to back electron/hole recombination across the space charge barrier on ms–s timescales, in kinetic competition with water oxidation. This loss necessitates the additional application of a 600–700 mV anodic potential for photocurrent (water oxidation) generation (see Fig. 6a). This implies that for unmodified BiVO_4_ photoanodes, a strongly anodic potential, resulting in a wide space charge layer and strong band bending, is required to provide a sufficient barrier to prevent back electron/hole recombination.^20^ For the CoPi-modified BiVO_4_ photoanode, the significantly smaller difference between the potential dependence of *φ*_TAS2_ (*i.e.* charge separation yield) and photocurrent implies that back electron/hole recombination losses are less severe. We note that the potential for the onset of long-lived holes in unmodified and CoPi-modified BiVO_4_ are similar (both ∼0.2 *V*_RHE_), suggesting that the flat-band potential is largely unaffected by CoPi deposition. This is consistent with studies of cobalt-modified BiVO_4_ (ref. 12) and α-Fe_2_O_3_ (ref. 14) photoanodes by other groups. Additionally, the amplitude of the long-lived holes is only slightly increased by CoPi (Fig. 4d) indicative of only minor retardation of sub-ms electron/hole recombination and indicating that the yield of hole transfer to the photoanode surface is largely independent of CoPi surface modification. These results suggest that the main effect of CoPi on BiVO_4_ photoanodes is to significantly reduce recombination of bulk electrons with surface accumulated holes.

### 4.2 Kinetic model for photoanode function

**Scheme 1:**
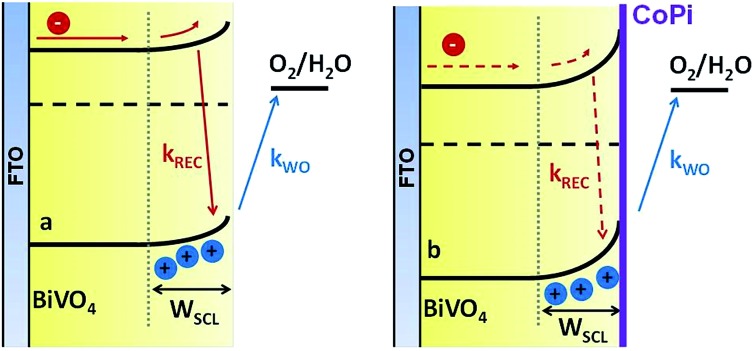
Schematic presentation of charge carrier dynamics considered in our kinetic model of water oxidation, illustrating the kinetic completion between water oxidation (*k*_WO_) and back electron/hole recombination (*k*_REC_), following photoexcitation of undoped unmodified (a) and CoPi-modified (b) BiVO_4_ photoanodes. For the CoPi-modified film, these back electron/hole recombination losses are significantly suppressed.

We now consider a simple kinetic model for water oxidation focused on the kinetic competition between water oxidation and back electron/hole recombination, as shown in Scheme 1. We have previously applied this kinetic model to unmodified BiVO_4_ and α-Fe_2_O_3_ photoanodes.^20,29^ Assuming 100% faradaic efficiency of water oxidation during the transient absorption measurements, the relative water oxidation yield, *φ*_WO_ (V), and back electron/hole recombination yield, *φ*_REC_ (V), can be calculated from:4
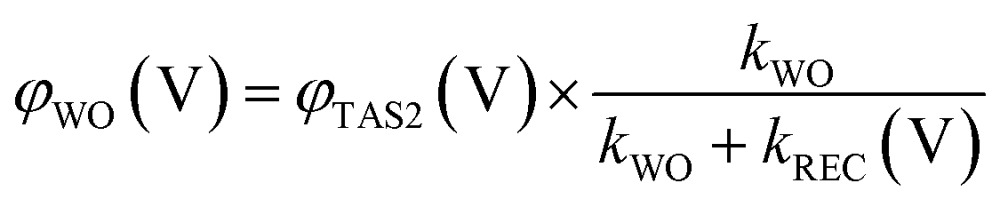

5
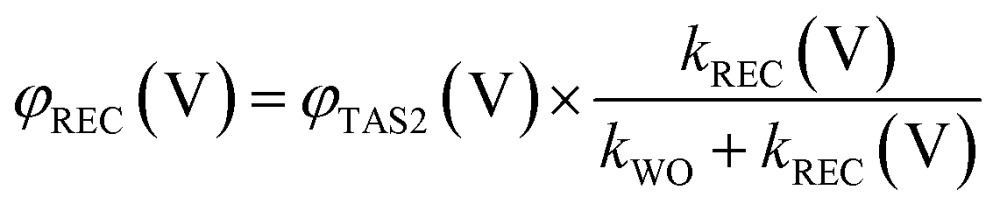

6
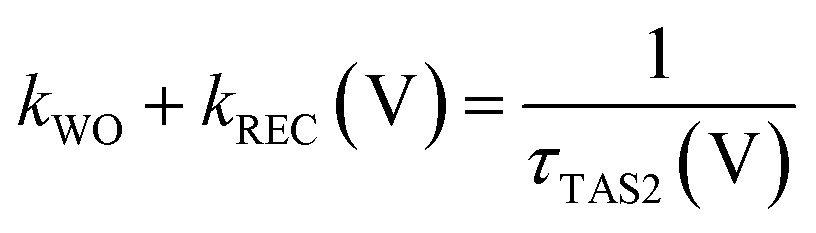
where *φ*_TAS2_ (V) and *τ*_TAS2_, are the amplitudes and lifetimes of the slow transient absorption decay phase, and therefore proportional to the yield and lifetimes of holes transferred to the photoanode surface, as shown in Fig. 4d; *k*_WO_ and *k*_REC_ (V) are the water oxidation and back electron/hole recombination rate constants, respectively. The water oxidation rate constant is assumed, as previously,^20^ to be potential independent, and is determined directly from *τ*_TAS2_ in the limit of strong anodic bias, where back electron/hole recombination does not occur.

The water oxidation yield *φ*_WO_ (V) calculated from eqn (4) using the data shown in Fig. 4 is shown in Fig. 6 (filled circles/squares). For both the unmodified and CoPi-modified BiVO_4_ photoanodes, the calculated water oxidation yield is in excellent agreement with the measured photocurrent density, demonstrating the effectiveness of this simple kinetic model.

The yield of back electron/hole recombination *φ*_REC_ (V), as quantified from eqn (5) from our transient absorption data, is shown in Fig. 5 (red symbols). This is overlaid with the charge lost through back electron/hole recombination as determined from integration of photocurrent transients (black lines). For both photoanodes, there is an excellent agreement between our transient absorption and transient photocurrent assays of the losses due to back electron/hole recombination, providing further support for the validity of our analyses. Also clear from Fig. 5, the losses of charges due to back electron/hole recombination are greatest around the photocurrent onset potential, *i.e.* 0.4 *V*_RHE_ for CoPi-modified BiVO_4_ and 0.7 *V*_RHE_ for unmodified BiVO_4_. This has previously been observed for α-Fe_2_O_3_ and undoped BiVO_4_ photoanodes.^20,29^ With increasing anodic potential, the width of the space charge layer increases, so more photogenerated holes reach the photoanode surface, where they accumulate.^20^ However at modest anodic bias the band bending associated with this space charge layer is insufficient to prevent back recombination of these surface holes with bulk electrons. As such, prior to the photocurrent onset, losses due to back electron/hole recombination increase with increasing anodic bias. At larger anodic potentials, the band bending becomes large enough so that the kinetics of this back recombination are slower than water oxidation, resulting in a reduction in back electron/hole recombination losses and the concomitant onset of water oxidation photocurrent. The time constants of back electron/hole recombination and water oxidation underlying this behavior, determined using the model above, are shown in Fig. S3,† which result in the observed peak of losses due to back electron/hole recombination at the photocurrent onset potential, as shown in Fig. 5. Our analysis shows the origin of the cathodic shift of the photocurrent onset potential following CoPi deposition on BiVO_4_. It appears that CoPi deposition does not substantially change the potential dependence or efficiency of hole transport to the BiVO_4_ surface. For both photoanodes, the onset potential for the generation of long-lived (ms–s) holes is ∼0.2 *V*_RHE_, close to the flat band potential for BiVO_4._ However CoPi deposition does reduce the additional potential required to turn off back electron/hole recombination, resulting in the observed cathodic shift in photocurrent generation.

Under sufficiently strong anodic bias, back electron/hole recombination is suppressed for both unmodified and CoPi modified photoanodes; under these conditions *τ*_TAS2_ corresponds directly to the time constant for water oxidation. It is apparent from the data in Fig. 4 that this time constant is similar for both photoanodes (1.4 s and 1.6 s without and with CoPi deposition). It can thus be concluded that the rate constant for water oxidation by BiVO_4_ holes is independent of CoPi. This time constant was also found to be independent of laser excitation density (see ESI Fig. S4 and S5†). It is therefore apparent that CoPi does not accelerate water oxidation on BiVO_4_ photoanodes. This is consistent with our previous transient absorption studies of CoPi-modified α-Fe_2_O_3_,^4,5^ and also an intensity-modulated photocurrent spectroscopy study which concluded that cobalt species do not increase the reaction rate of water oxidation on a cobalt-treated α-Fe_2_O_3_ photoanode.^15^ If anything, CoPi deposition appears to result in a small retardation of water oxidation (from 1.4 to 1.6 s), most probably due to the reduced accessibility of water to the BiVO_4_ surface following CoPi deposition. We note that these data do not mean that CoPi is not oxidized under steady-state irradiation at anodic bias (resulting for example from a low quantum yield of hole transfer from BiVO_4_ to CoPi) but only that such oxidation, if present, is relatively unimportant in explaining the cathodic shift in photocurrent onset potential following CoPi deposition. A detailed study of this issue, employing continuous illumination, is ongoing and will be reported elsewhere. We also note that it has been suggested that on α-Fe_2_O_3_ photoanodes, thicker than optimum CoPi layers may result in a higher proportion of water oxidation proceeding from oxidized CoPi.^6^ The study herein employed the thickness found to give optimum performance. Notwithstanding these caveats, the data reported herein imply that, for the optimized CoPi layer thickness employed, any such oxidized CoPi, if present, does not contribute strongly to the enhanced water oxidation photocurrent. Instead, for BiVO_4_ modified by a CoPi overlayer, the improved photocurrent onset potential and higher photocurrent density can be explained by efficient suppression of back electron/hole recombination.

Previously we have observed significant retardation of electron/hole recombination following CoPi-modification of α-Fe_2_O_3_ photoanodes.^4,5^ These previous studies did not distinguish between the impact of CoPi deposition on bulk electron/hole recombination (*i.e.* the efficiency of hole transport to the surface) or on back electron/hole recombination. We also note that back electron/hole recombination losses were less prominent for these hematite photoelectrodes compared to the BiVO_4_ photoanodes reported herein. This is most probably associated with the higher doping density and faster bulk recombination losses in the hematite photoanodes. Nevertheless our previous results are consistent with the results presented herein, namely that the primary effect of CoPi deposition is the retardation of recombination losses rather than acceleration of water oxidation.

Our transient absorption study reported herein indicates that the charge separation (*i.e.* fraction of holes reaching the BiVO_4_ surface) is not significantly improved in the presence of CoPi overlayer, as evidenced by the little improved initial amplitude of long-lived holes (milliseconds to seconds) in BiVO_4_ photoanodes following CoPi modification. Rather our results demonstrate that the primary function of CoPi overlayers is retardation of recombination. This is in broad agreement with previous PEC studies^8,10^ that indicated that recombination within the space charge layer is reduced in CoPi-modified BiVO_4_ photoanodes. More specifically, and in contrast to these PEC studies, our results from CoPi-modified BiVO_4_ indicate that such lower recombination losses result primarily from an inhibition of back electron/hole recombination *within* the BiVO_4_ photoelectrode, rather than across the BiVO_4_/CoPi interface.

The mechanism of how CoPi deposition reduces the losses due to back electron/hole recombination within the BiVO_4_ is not clear. Previously we suggested that reduced recombination losses in CoPi modified α-Fe_2_O_3_ photoanodes may result from enhanced band bending and space charge layer formation induced by CoPi.^4,5^ We note that the space layer depth in these doped α-Fe_2_O_3_ photoanodes^29^ (∼8 nm at 1.23 *V*_RHE_) is substantially narrower than in the undoped BiVO_4_ studied herein^20^ (∼90 nm at 1.23 *V*_RHE_). The small increase in the long-lived hole amplitude (Fig. 4d) and photocurrent (Fig. 1 and S2 in the ESI†) in CoPi-modified BiVO_4_ suggests that the width of the space charge layer is marginally increased in the presence of CoPi overlayers. Instead, the significant reduction of back electron/hole recombination may be caused by increased electric field strength associated with the electrostatics of the BiVO_4_/CoPi interface, although detailed understanding of this point is beyond the scope of this study.

## 5. Conclusion

Transient absorption spectroscopy and transient photocurrent measurements were employed to characterize the dynamics of photogenerated holes in CoPi-modified BiVO_4_ photoanodes. The competition between water oxidation and electron/hole recombination was quantified using a simple kinetic model. The photoelectrochemical results confirm that the water oxidation performance of BiVO_4_ photoanodes can be improved by CoPi deposition, resulting in a cathodic shift in the photocurrent onset potential. However, transient absorption measurements, which allow direct monitoring of photogenerated holes, and transient photocurrent studies indicate that this improvement is not primarily due to the catalytic function of CoPi on the BiVO_4_ surface, under the measurement conditions employed. Rather, the cathodic shift in onset potential results from suppression of recombination of surface-accumulated holes with bulk electrons (back electron/hole recombination). This allows more holes to contribute to water oxidation. These results emphasize the key role of recombination losses in limiting photoanode performance, and how these losses can be reduced by surface modification.
