# Supplementary material for: Efficient suppression of back electron/hole recombination in cobalt phosphate surface-modified undoped bismuth vanadate photoanodes
Source: J Mater Chem A Mater. 2015 Sep 21;3(41):20649–57. doi: 10.1039/c5ta05826k (PMC4894069; doi:10.1039/c5ta05826k)
Supplement: Supplementary file 1 [file TA-003-C5TA05826K-s001.pdf]

Electronic Supplementary Material (ESI) for Journal of Materials Chemistry A.
This journal is © The Royal Society of Chemistry 2015

Efficient Suppression of Back Electron/Hole Recombination in Cobalt Phosphate Surface-Modified Undoped Bismuth Vanadate Photoanodes

Yimeng Ma,[a] Florian Le Formal,[a,b] Andreas Kafizas,[a] Stephanie R. Pendlebury,[a] James R. Durrant[a]*

[a] Department of Chemistry, Imperial College London, South Kensington Campus, London, SW7 2AZ, United Kingdom. E-mail: j.durrant@imperial.ac.uk

[b] Laboratory for Molecular Engineering of Optoelectronic Nanomaterials, Institute of Chemical Sciences and Engineering, École Polytechnique Fédérale de Lausanne (EPFL), Station 6, CH H4 565, Lausanne 1015, Switzerland

1. XRD and SEM characterizations of CoPi-modified and unmodified $BiVO_4$ photoanodes

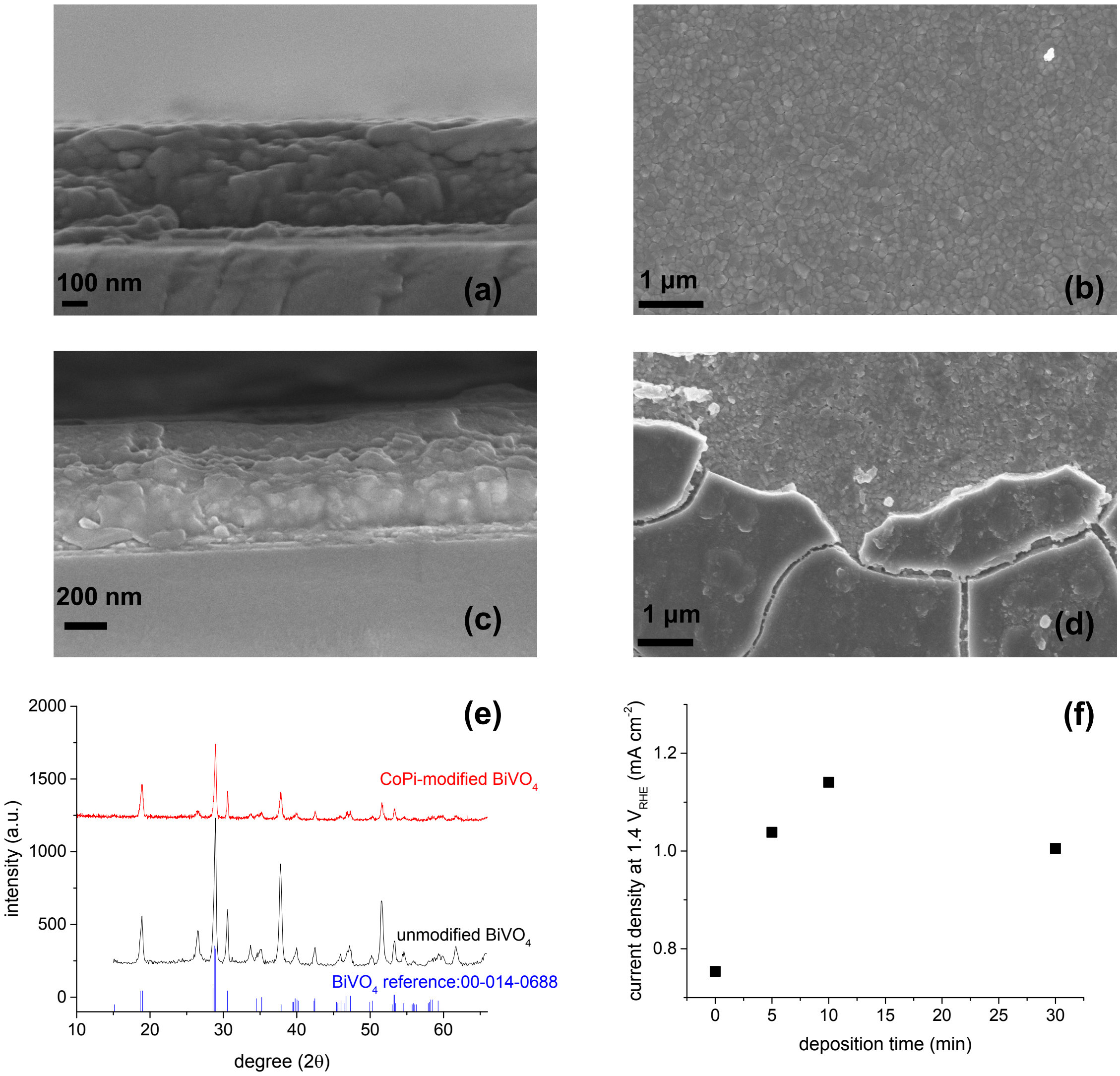

Fig. S1 (a) – (d) SEM images of unmodified (cross-sectional: (a); top: (b)) and CoPi-modified (cross-sectional: (c); top: (d)) $BiVO_4$ photoanodes. (e): XRD patterns of CoPi-modified (red) and unmodified (black) $BiVO_4$ photoanodes. Reference (blue) of monoclinic $BiVO_4$: 00-014-0688. (f) The photocurrent of CoPi-modified $BiVO_4$ photoanodes recorded at 1.4 V_{RHE} as a function of CoPi deposition time.

2. Photoelectrochemical characterization of CoPi-modified $BiVO_4$ photoanodes

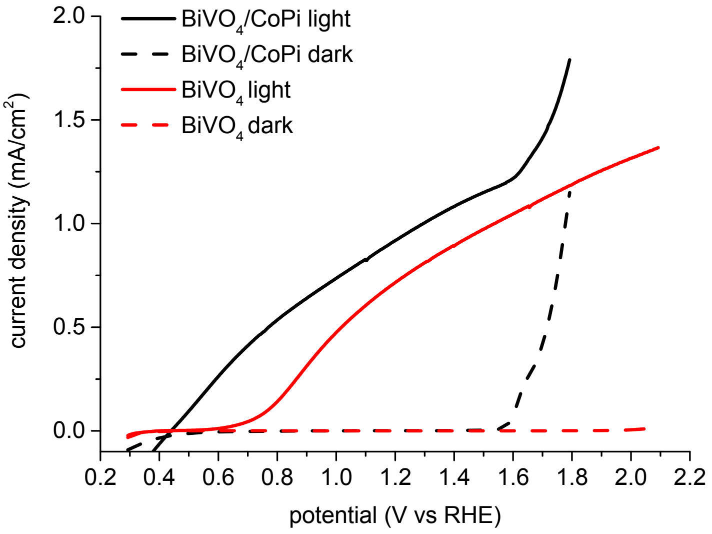

Fig. S2 Current densities of an unmodified $BiVO_4$ (red) and a CoPi surface modified $BiVO_4$ (black) photoanode as a function of applied potential vs RHE measured in dark (dashed lines) and light (solid lines). Scan rate: 10 mV s^{-1}.

3. Fit results of transient absorption decays in unmodified and CoPi/$BiVO_4$ photoanodes under applied potentials

Table S1 Fit results of transient absorption decays of unmodified and CoPi/$BiVO_4$ photoanodes as a function of applied potential using a combination of power law and single exponential function shown in Equation 2 in the main paper

potential (V vs RHE)	CoPi-modified $BiVO_4$				unmodified $BiVO_4$			
	a	b	ϕ_{TAS2} (mΔOD)	τ_{TAS2} (s)	a	b	ϕ_{TAS2} (mΔOD)	τ_{TAS2} (s)
1.6	-	-	-	-	3.1E-06	-0.24	0.036	1.4
1.5	-	-	-	-	3.1E-06	-0.25	0.036	1.4
1.4	3.7E-07	-0.41	0.042	1.6	3.0E-06	-0.23	0.035	1.2
1.2	1.1E-06	-0.32	0.040	1.6	1.1E-06	-0.33	0.032	1.0
1	2.5E-07	-0.44	0.033	1.6	3.6E-06	-0.23	0.028	0.9
0.8	9.0E-07	-0.35	0.026	1.5	2.7E-06	-0.25	0.019	0.8
0.6	9.3E-07	-0.35	0.019	1.3	1.0E-06	-0.35	0.013	0.2
0.4	1.0E-06	-0.31	0.010	0.2	3.5E-06	-0.22	0.012	0.1
0.2	1.9E-08	-0.35	0.003	0.02	1.2E-06	-0.22	0.004	0.01

4. Time constants of the slow phase (ms–s) in unmodified and CoPi-modified $BiVO_4$ photanodes

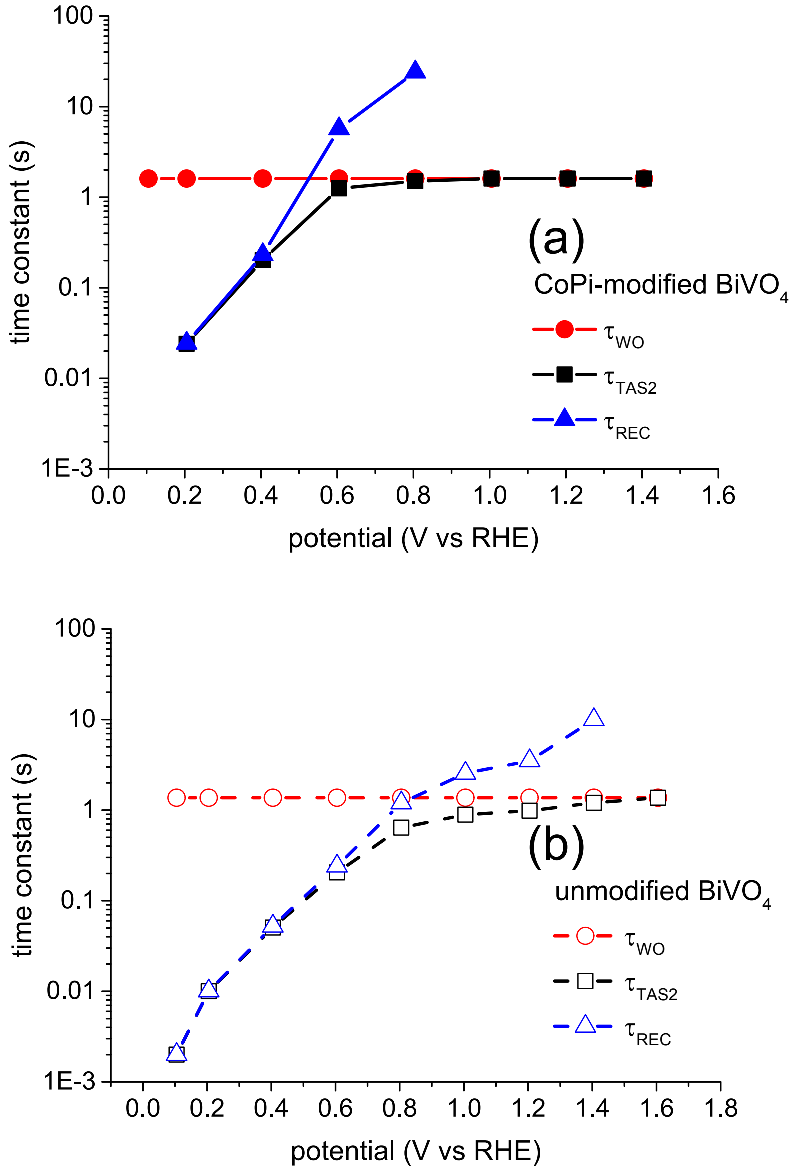

Fig. S3 Comparison of time constants of water oxidation (τ_{WO}, red), back electron/hole recombination (τ_{REC}, blue) and total transient absorption decay on ms-s timescales (τ_{TAS2}, black) obtained from Equation 2 and 6 in the main paper. (a): CoPi-modified $BiVO_4$; (b): unmodified $BiVO_4$.

5. Kinetics of photogenerated holes in CoPi-modified $BiVO_4$ as a function of excitation intensity

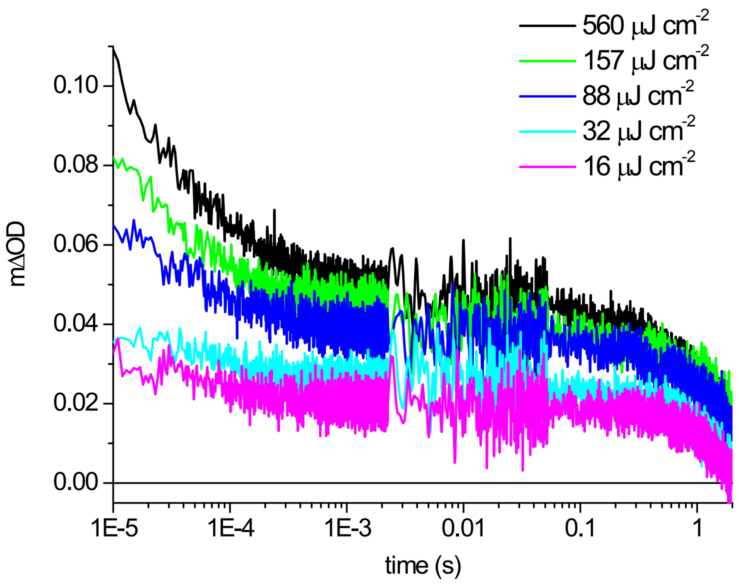

Fig. S4 Transient absorption decays of a CoPi-modified $BiVO_4$ photoanode measured as 1.2 V_{RHE} as a function of excitation intensity.

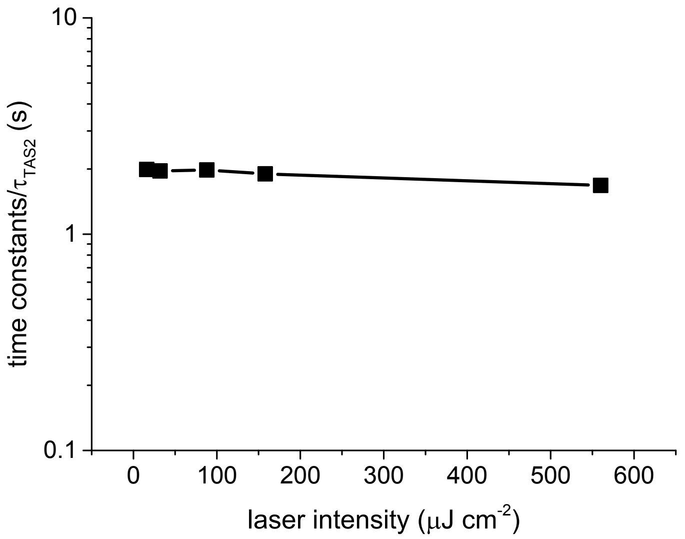

Fig. S5 Slow phase time constants (τ_{TAS2}) of transient absorption decays at 1.2 V_{RHE} as a function of excitation intensity in a CoPi-modified $BiVO_4$ photoanode. The time constants are obtained from fitting the transient absorption decays in Fig. S4 using Equation 2 in the main paper.
